# Improving MGMT methylation status prediction of glioblastoma through optimizing radiomics features using genetic algorithm-based machine learning approach

**DOI:** 10.1038/s41598-022-17707-w

**Published:** 2022-08-04

**Authors:** Duyen Thi Do, Ming-Ren Yang, Luu Ho Thanh Lam, Nguyen Quoc Khanh Le, Yu-Wei Wu

**Affiliations:** 1 Graduate Institute of Biomedical Informatics, College of Medical Science and Technology, Taipei Medical University, 15th Floor, No. 172-1, Keelung Rd., Sect. 2, Da-an District, Taipei, 106 Taiwan, ROC; 2 Department of Electrical Engineering, National Taiwan University of Science and Technology, Taipei, Taiwan, ROC; 3 International Master/Ph.D. Program in Medicine, College of Medicine, Taipei Medical University, Taipei, Taiwan, ROC; 4 Professional Master Program in Artificial Intelligence in Medicine, College of Medicine, Taipei Medical University, 19th Floor, No. 172-1, Keelung Rd., Sect. 2, Da-an District, Taipei, 106 Taiwan, ROC; 5 Research Center for Artificial Intelligence in Medicine, Taipei Medical University, Taipei, Taiwan, ROC; 6 Translational Imaging Research Center, Taipei Medical University Hospital, Taipei, Taiwan, ROC; 7 Clinical Big Data Research Center, Taipei Medical University Hospital, Taipei, Taiwan, ROC

**Keywords:** Biomarkers, Cancer imaging, Image processing

## Abstract

O6-Methylguanine-DNA-methyltransferase (MGMT) promoter methylation was shown in many studies to be an important predictive biomarker for temozolomide (TMZ) resistance and poor progression-free survival in glioblastoma multiforme (GBM) patients. However, identifying the MGMT methylation status using molecular techniques remains challenging due to technical limitations, such as the inability to obtain tumor specimens, high prices for detection, and the high complexity of intralesional heterogeneity. To overcome these difficulties, we aimed to test the feasibility of using a novel radiomics-based machine learning (ML) model to preoperatively and noninvasively predict the MGMT methylation status. In this study, radiomics features extracted from multimodal images of GBM patients with annotated MGMT methylation status were downloaded from The Cancer Imaging Archive (TCIA) public database for retrospective analysis. The radiomics features extracted from multimodal images from magnetic resonance imaging (MRI) had undergone a two-stage feature selection method, including an eXtreme Gradient Boosting (XGBoost) feature selection model followed by a genetic algorithm (GA)-based wrapper model for extracting the most meaningful radiomics features for predictive purposes. The cross-validation results suggested that the GA-based wrapper model achieved the high performance with a sensitivity of 0.894, specificity of 0.966, and accuracy of 0.925 for predicting the MGMT methylation status in GBM. Application of the extracted GBM radiomics features on a low-grade glioma (LGG) dataset also achieved a sensitivity 0.780, specificity 0.620, and accuracy 0.750, indicating the potential of the selected radiomics features to be applied more widely on both low- and high-grade gliomas. The performance indicated that our model may potentially confer significant improvements in prognosis and treatment responses in GBM patients.

## Introduction

Glioblastoma multiforme (GBM), one of the most common brain tumors, accounts for approximately 45% of all malignant types of central nervous system tumors and is more likely to develop as primary (de novo) GBM^1^. Despite some advances in standard multimodal treatment, including surgical resection followed by adjuvant chemoradiotherapy and adjuvant chemotherapy, the median survival of patients remains pretty low, at only 14–16 months^2,3^. GBM is considered a deadly disease with a poor prognosis due to its biological complexity, high frequency of chemotherapeutic resistance, and frequent recurrence after surgical treatment^4^.

The standard chemotherapeutic agent for GBM treatment is temozolomide (TMZ), an alkylating drug that makes cells more sensitive to radiation^5^. This agent exerts its cytotoxic effects through methylating O6-methylguanine, which in turn causes DNA damage leading to cell death^6,7^. Thus, a major obstacle to the successful treatment of GBM is inherent and/or acquired chemoresistance to TMZ regulated by an enzyme called O6-methylguanine-DNA methyltransferase (MGMT), which is a highly evolutionarily conserved DNA repair enzyme that removes alkylated guanine residues at the DNA level, thereby antagonizing the effects of alkylating therapeutic agents^8,9^. Since methylation of CpG islands in the MGMT promoter region leads to suppression of MGMT transcription^10,11^, it could be a potential predictive biomarker for TMZ resistance and poor progression-free survival. It is thus important to identify the MGMT methylation status to have an accurate treatment strategy and improve success rates for GBM treatment.

Although molecular techniques using surgical specimens are considered as reference standards for evaluating the MGMT methylation status, determining this epigenetic modification by a methylation-specific polymerase chain reaction often requires a large volume of tissue sample and a strict sample cryopreservation procedure^12^, while other techniques such as activity assays, immunohistochemistry, and methylation chip analysis have technical constraints^13^. In addition, the possibility of incomplete biopsy sampling, high prices for detection, and the high complexity of intralesional heterogeneity render these invasive techniques less useful in hospitals^14^.

Recently, experts’ interest has shifted toward using non-invasive techniques such as radiomics to discover links between clinical symptoms and genetic characteristics^15^. Radiomics has been used to quantitatively extract and analyze noninvasive medical imaging features, including intensity distributions, textural heterogeneity patterns, spatial relationships, and many other characteristics^16,17^. In recent years, some radiological research has developed radiomics models for predicting survival rates^16^, distant metastasis^18^, and characterizations of molecular characteristics^19^. As the MGMT methylation status is considered an important prognostic biomarker for guiding GBM treatment decisions, several computational models were also developed to preoperatively predict the MGMT methylation status based on magnetic resonance imaging (MRI)^20–22^. In a recent study, Le et al. proposed a radiomics-based eXtreme Gradient Boosting (XGBoost) model that achieved relatively high performance for predicting the MGMT promoter methylation status, with an accuracy of 88.7% and an area under the receiver operating characteristics curve (AUC) of 0.896^23^.

Inspired by this previous work of Le et al.^23^, we proposed a hybrid machine learning feature selection model in this paper to obtain the most informative radiomics feature set and meticulously evaluate its capability of accurately classifying MRI images into methylated and unmethylated ones. The proposed feature selection approach consisted of two steps: the XGBoost algorithm was first employed to extract features relevant to the MGMT methylation status; the selected feature set was then fed into a genetic algorithm (GA)-based wrapper model, in which the radiomics feature set with the best prediction power were selected under schemes similar to and inspired by natural selection in order to identify the “fittest” set of features for predicting MGMT methylation status. The performance evaluations of the GA-based approach also revealed that our GA-based hybrid model achieved a better performance for detecting the MGMT methylation status compared to other models, indicating the potential of the proposed hybrid approach in effectively predicting MGMT methylation status.

## Materials and methods

**Figure 1:**
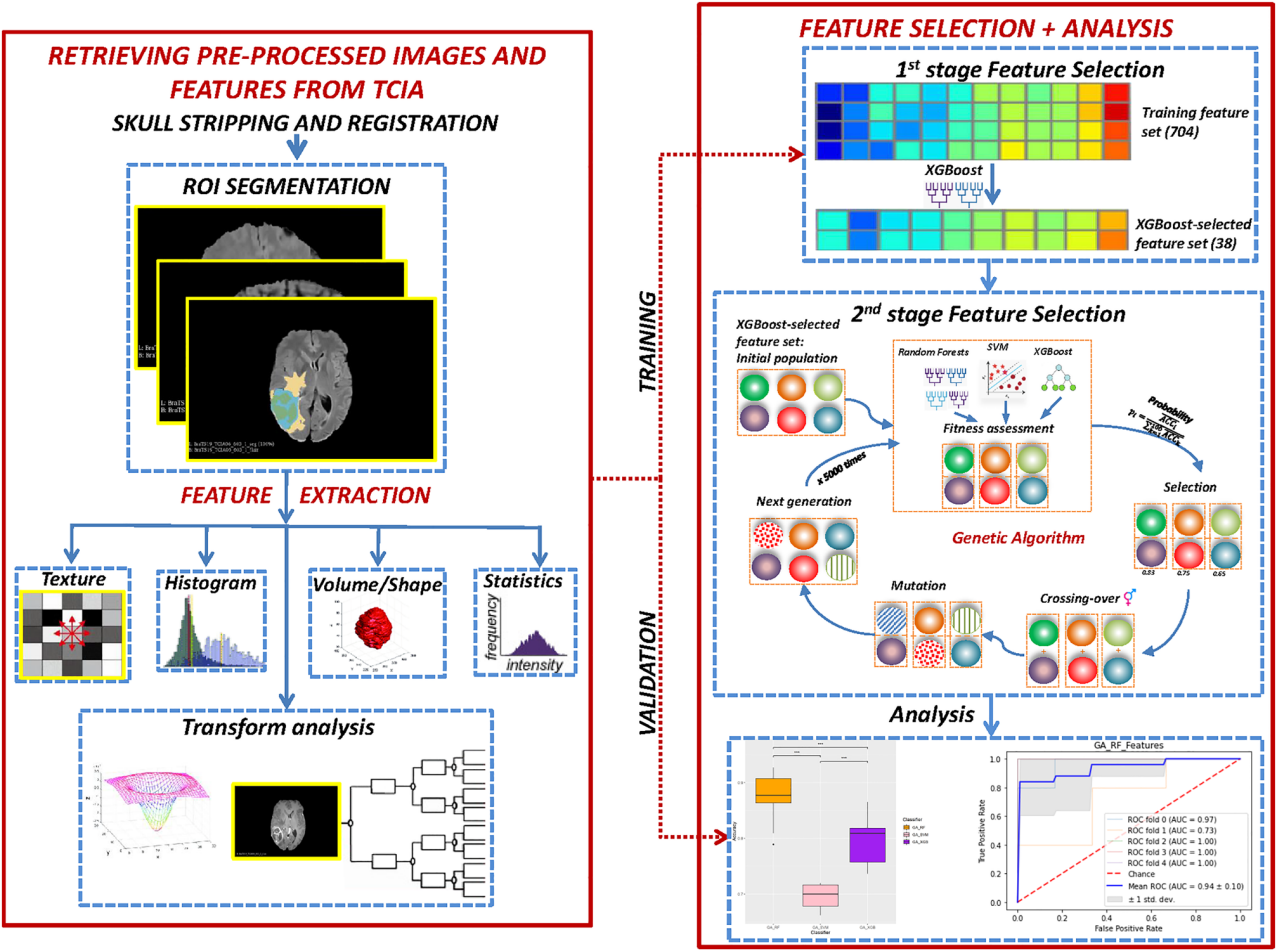
The overall feature selection steps. The left part demonstrates the pre-processing and segmentation steps while the right part list the two-stage feature selection procedure. The extracted feature set is then evaluated for its efficacy.

Our proposed method for building an effective model for predicting MGMT methylation status consists of the following steps. Upon downloading the pre-processed and segmented multimodal MRI (mMRI) features from the TCIA public database, a two-stage radiomics feature selection approach was conducted on the mMRI feature set to identify the most informative features for MGMT methylation status classification purpose. The procedure for conducting feature selection and evaluating the efficacy of the features was illustrated in Fig. 1.

### Data source and collection

The pre-processed and segmented multimodal MRI features from The Cancer Genome Atlas (TCGA)-GBM^24^ collections were downloaded from Bakas et al.^25^. Only data entries with MRI modalities of at least one of the following types were selected: T1-weighted pre-contrast (T1), T1-weighted post-contrast (T1-Gd), T2, and T2-FLAIR (fluid-attenuated inversion recovery). As a result, 53 GBM patients were included in this study. Totally 704 radiomics features were obtained from Bakas et al.^25^ and can be classified into seven categories. The categories include (1) first-order statistical features (intensity), (2) volumetric features^26^, (3) textural features describing the statistical relationship between image voxels (e.g., gray-level co-occurrence matrix (GLCM)^27^, gray-level run length matrix (GLRLM)^28,29^, gray-level size zone matrix (GLSZM)^30^, neighboring gray-tone difference matrix (NGTDM)^31^, and wavelet-based features^32^, (4) histogram-based features^33^, (5) morphologic features^34^, (6) spatial features, and (7) glioma diffusion properties extracted from glioma growth models^35,36^.

### Radiomics feature selection and genetic algorithm (GA)

Feature selection is an important task in eliminating noisy variables, keeping only features that are helpful in the classification tasks. In this study, we performed a two-stage radiomics feature selection using XGBoost and a genetic algorithm (GA) to discover the most appropriate subset of features that contributed to improving the MGMT methylation status prediction. The TCGA-GBM dataset with 704 radiomics features was used as the input. In the first feature selection stage, we used the XGBoost classification model (objective = “binary:logistic”, booster = “gbtree”, eta = 0.3, gamma = 0, max depth = 6, lamda = 1, binary = “hinge”) to preliminarily determine features that may be important for our model. The gain score was utilized in determining feature importance. The XGBoost-selected feature set was then fed into the next stage of feature selection using the GA. By subjecting the feature selection process to an evolutionary-based mutational model, the GA could optimize the radiomics feature subset for highly effective MGMT methylation status prediction.

**Figure 2:**
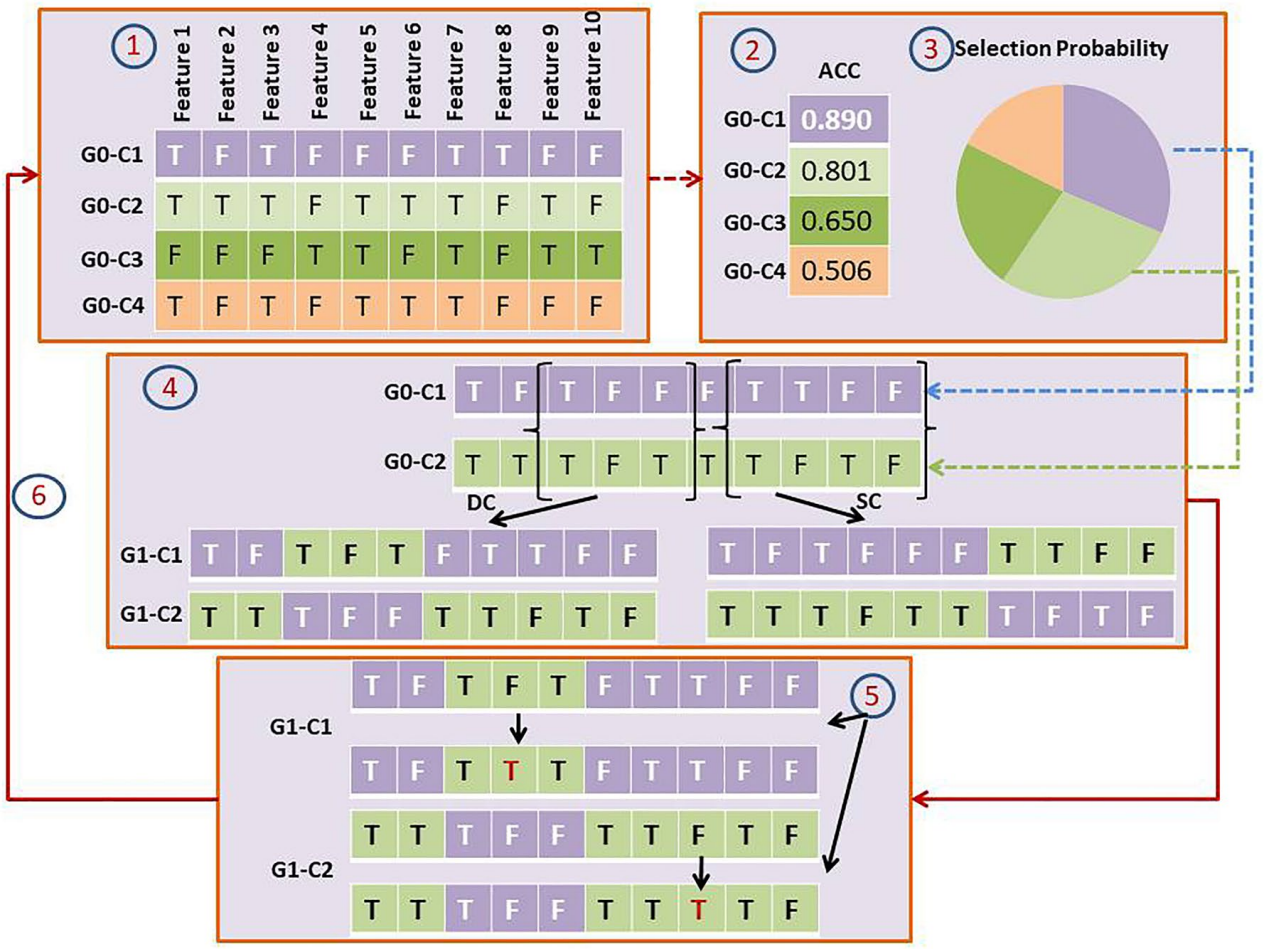
The Genetic Algorithm workflow. The steps are: (1) Generation of the initial population of solutions; (2) Evaluation of fitness values of each solution within the population; (3) The “mating” process of the solution, in which the probability of a solution to be selected is proportional to the estimated fitness value; (4) The random designation of crossover points on each vector of solution during the “mating” process. SC and DC stand for Single- and Double-Crossover, respectively; (5) The introduction of random mutations on the crossover-ed solution vectors; (6) The replacement of the entire population by daughter solutions.

The detailed workflow of the GA is as follows. An example that depicts how GA works can be seen in Fig. 2.

1. Generation of the initial population: this stage is to create a set of solutions (initial population), and each solution is one “chromosome (termed GA-chromosome hereafter)” indicating the inclusion or exclusion of the radiomics features. Each potential feature has an equal probability to be included (1) or excluded (0) from each GA-chromosome, resulting in a vector of 0 and 1 bits (the length of the vector was the number of to-be evaluated features). Initially, the algorithm randomly generated* x* GA-chromosomes for one population in each generation, in which the length of each GA-chromosome was the total number of XGBoost-selected features. The value of *x* was set to 100.
2. Fitness assessment: In this stage, the suitability (i.e. whether the combination of selected features results in good prediction) of each GA-chromosome in a population is evaluated based on the “fitness values” representing the ability to predict MGMT methylation statuses. The idea of a wrapper model, in which the fitness was defined as the accuracy of a machine learning model built on the selected feature set, was incorporated in determining fitness values. Several ML models, including support vector machine (SVM), random forest (RF), and XGBoost were evaluated to choose the most suitable model for the prediction purpose. More detailed description of the ML models will be explained in more detail in the Classification Algorithms subsection. Repeated five-fold cross validation was utilized for evaluating the model accuracy. In addition, elitism was applied in our GA to preserve the best feature set from generation to generation, in which two GA-chromosomes with the highest accuracy were copied into the next generation.
3. Selection of Parents: The individual genomes were selected for mating and crossover purposes as part of the GA algorithm, in which the probability that an individual genome was selected is proportional to its fitness value. The selection probability was calculated by the following formula:
  1 $${p}_{i}=\frac{\overline{{ACC }_{i}}}{{\sum }_{k=1}^{100}\overline{{ACC }_{k}}}$$
  where *i* and $\overline{ACC}$ respectively denote the *i*th GA-chromosome and the mean accuracy evaluated by the “fitness function” for the *i*th GA-chromosome. This step allows fit individuals to be selected with a higher probability while still giving some chances for good characteristics of less fit GA-chromosomes to be passed to the next generation.
4. Crossover: In each generation, selected parent solutions exchange their GA-chromosome segments to create new individuals. The “cross-over” points (*n* and *m* chiasmata) in each pair of parent chromosomes were sampled within the GA-chromosome. A single-(SCs) or double-crossover (DCs) were then conducted at the chiasmata, interchanging chromosome content between the two selected parent chromosomes. The crossover rate was set to 0.8. This crossover procedure was applied repeatedly until *x* offspring were generated.
5. Mutation: To protect against stagnation or in-breeding and maintain the diversity of GA populations, mutations are introduced to each child chromosome of a population. Inspired by the state-of-the-art GA algorithm developed by Her et al.^37^, a high mutation probability of 0.05 was established, and the mutation points were integer values randomly sampled from the genome vector. The (1) or (0) values representing either the inclusion or exclusion of the randomly sampled features were then flipped.
6. Population replacement: A new generation consisting of child chromosomes generated from the aforementioned steps along with the two “elite” GA-chromosomes with highest scores were placed into the next generation and replaced the initial population. This process was repeated 5000 times for a GA run.

### Classification algorithms

ML techniques are becoming increasingly popular in radiomics studies for their ability to handle high-dimensional features and their robustness in capturing complex interactions among features themselves and between feature combinations for building effective prognostic/predictive models. In this study, supervised ML models including RF, XGBoost, and SVM were used to conduct the binary classification between MGMT methylated and unmethylated classes in GBM patients. The RF and XGBoost algorithms are ensemble learning techniques that collect individual outcome predictions from numerous weak learners and select the final model based on the votes. On the other hand, SVM can identify the most effective hyperplane for discriminating different targets and is able to transform a non-linearly distributed feature space into a high-dimensional feature space. The parameters for the models were: SVM with the radial-basis kernel, RF with 100 trees, and XGBoost classifier with default settings. The Python language and Scikit-learn package (https://scikit-learn.org/) was used for the model development. These three models were incorporated into the GA, and the model with the highest performance was chosen for further analysis. The predictive accuracy of the training dataset was evaluated using the five-fold stratified cross-validation method.

### Performance assessment

The classification performances of the models were evaluated using sensitivity, specificity, and accuracy. The comparison between the three ML models used in this study was made based on the mean of running cross-validation 20 times and evaluated using Kolmogorov–Smirnov test. Data were analyzed using Scipy package (https://scipy.org/)^38^. In addition, values of the receiver operating characteristic (ROC) curve and area under the ROC curve (AUROC or AUC) were also calculated to evaluate the overall performance. The comparison against other methods was made by extracting the model performances from the published studies.

### Predicting MGMT status for low-grade glioma (LGG) dataset

The mMRI radiomics features extracted using the GA-based wrapper model from the TCGA-GBM dataset were also applied to the MGMT status prediction for low-grade glioma (LGG) multimodal MRI dataset, which was also pre-processed and segmented from The Cancer Genome Atlas TCGA-LGG^39^ by Bakas et al.^25^ The features extracted by conducting the GA wrapper model on GBM was applied directly to the LGG dataset without conducting further feature selection procedure. The model performances were again evaluated by sensitivity, specificity, accuracy, and ROC/AUC. The published XGBoost-F-score model^23^ was also applied on the LGG dataset to compare its performances against the proposed GA wrapper model.

## Results

### Radiomics feature selection and ML classification

**Figure 3:**
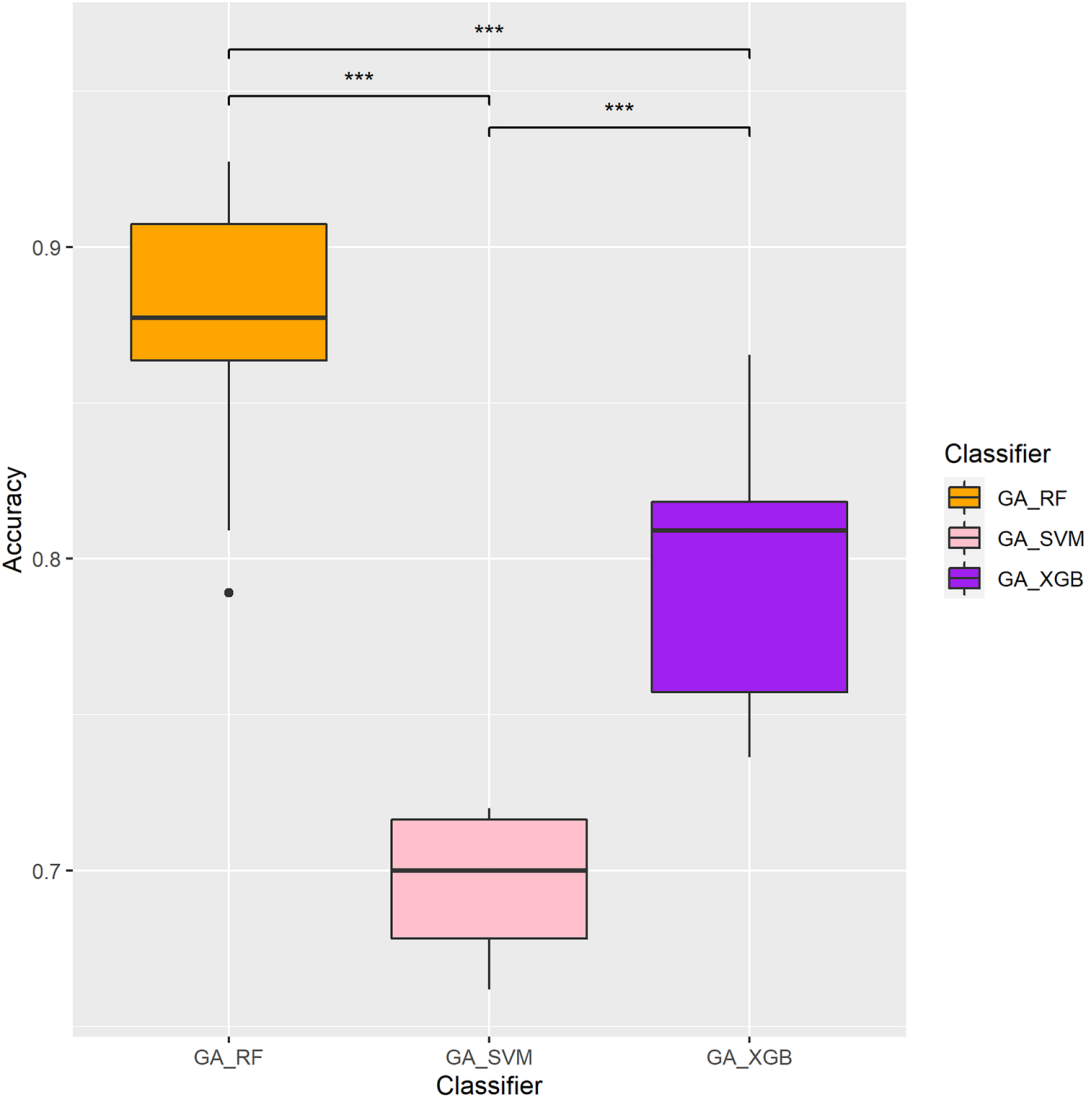
Performance evaluations and comparisons of different GA-incorporated models in predicting MGMT methylation statuses. Y-axis represents accuracy. Statistical significances evaluated by Kolmogorov–Smirnov test are represented by stars, in which three stars (***) indicate *p* < 0.001.

The numbers of MGMT methylated and unmethylated samples in the TCGA-GBM dataset were 26 and 27, respectively. The two-stage feature selection approach comprised of XGBoost followed by a GA algorithm for selecting the most representative features. Three different ML models (viz., RF, XGBoost, and SVM) were evaluated for their efficacy in the GA fitness wrapper model (see “Materials and methods”). As shown in Table 1, GA-RF (with RF incorporated in the GA; shown in bold font) achieved the best performance with a sensitivity of 0.894, specificity of 0.966, and accuracy of 0.925 at generation 2022, while GA-XGB yielded the second-best performance with a sensitivity of 0.889, specificity of 0.88, and accuracy of 0.889 at generation 3464 (see “Materials and methods” for details). In contrast, GA-SVM achieved a relatively low performance (sensitivity 0.720, specificity 0.454, and accuracy 0.678). Figure 3 illustrates the accuracy of each different ML-incorporated GA model evaluated by five-fold cross-validation for 20 times. One can see that the GA-RF model significantly outperformed the remaining models used in this research (*p* < 0.001; Kolmogorov–Smirnov test), leading to a more accurate prediction of the MGMT methylation status in GBM patients.

**Table 1:** Performance evaluations for different machine learning-incorporated genetic algorithm (GA) models on the GBM dataset.

Classifiers	No. of features	Sensitivity	Specificity	Accuracy
**GA-RF**	**18**	**0.894**	**0.966**	**0.925**
GA-XGB	18	0.889	0.88	0.889
GA-SVM	14	0.720	0.454	0.678

*RF* random forest, *XGB* XGBoost, *SVM* support vector machine.

Model with the best performance is indicated in bold font.

**Figure 4:**
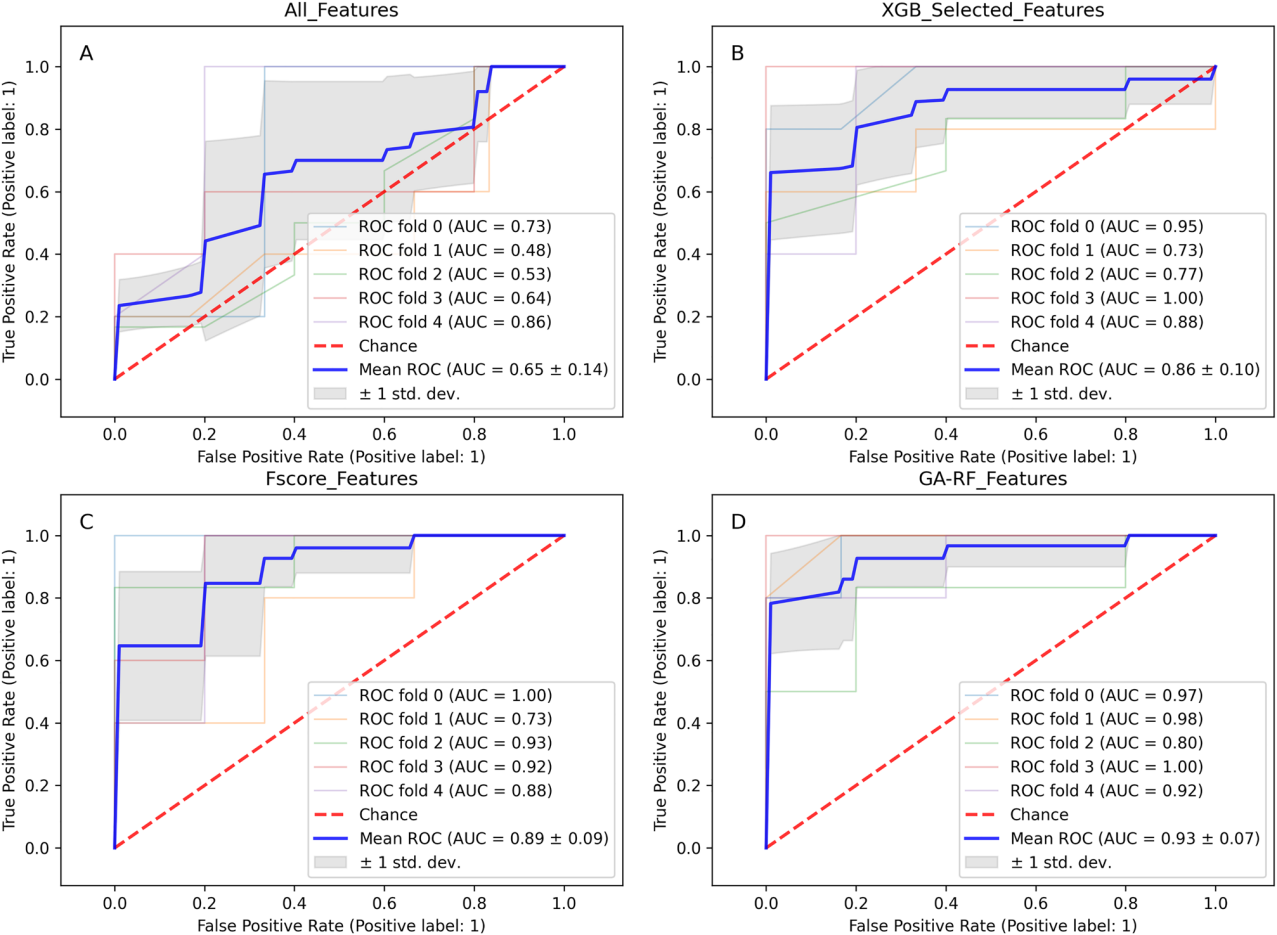
Receiver operating characteristic (ROC) curves of different feature sets as evaluated by the random forest (RF) algorithm. The feature sets are: (**A**) all 704 radiomics features; (**B**) 38 features selected by XGBoost; (**C**) the feature set selected by F-scores; and (**D**) the feature set selected by the genetic algorithm (GA)-RF algorithm.

**Figure 5:**
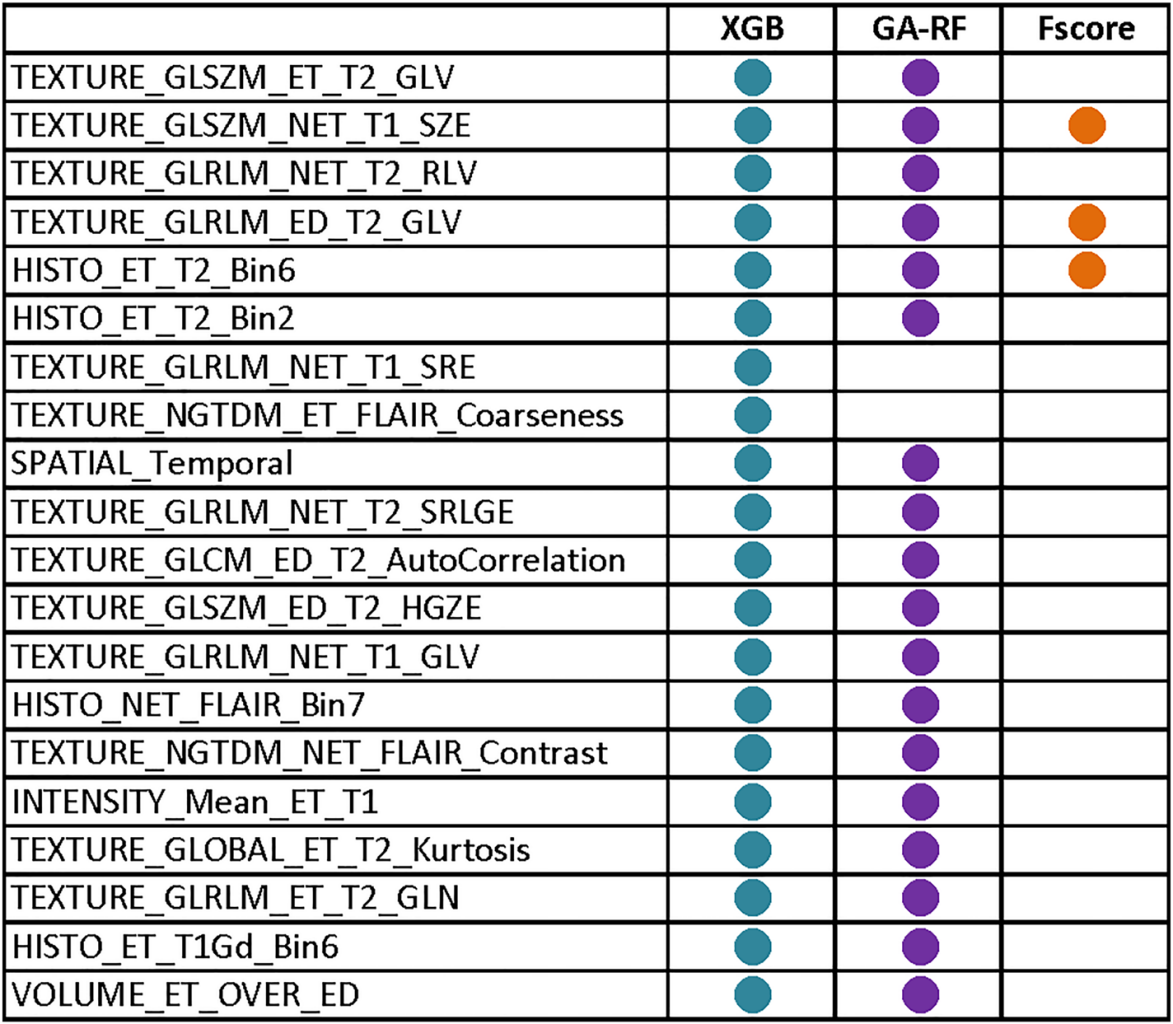
Common radiomics features selected by different methods. Solid circles represent the presence of certain features in each feature set.

Next, we compared the 22 GA-RF feature subset with feature sets selected by different methods, including all features (704 features), the subset of 25 features extracted by the XGBoost algorithm based on the gain score, and the set of radiomics features originated from the F-score feature selection analysis (conducted by Le et al.^23^). As shown in Fig. 4, the GA-RF feature set outperformed other feature sets with an outstanding AUC 0.93, indicating the capability of the GA-RF feature set in identifying MGMT methylation status from radiomics features. The comparison among different feature sets, as shown in Fig. 5, indicated that three radiomics features, including two textural features (TEXTURE_GLRLM_ED_T2_GLV and TEXTURE_GLSZM_NET_T1_SZE), and one histogram-based feature (HISTO_ET_T2_Bin6), were identified by both F-score feature selection method adopted by Le et al. and our GA-RF algorithm, hinting that these features might be key biomarkers for identifying MGMT-methylated tumors.

We also set to test the extracted GA-RF feature set to classify the MGMT methylation status for the low-grade glioma (LGG) dataset. We benchmarked the features extracted from the three GA-based machine learning algorithms from the GBM dataset on the LGG dataset. The XGBoost-F-score model developed by Le et al.^23^ was also evaluated. As shown in Table 2, the GA-RF algorithm (shown in bold font) outperformed other models with an accuracy of 0.750, a sensitivity of 0.78, and a specificity of 0.62. Even though the GA-SVM achieved the best sensitivity among the three models (0.84), its overall accuracy was the same as GA-XGB (0.71) and lower than GA-RF model due to their low specificity (0.23 and 0.46 for GA-SAM and GA-XGB, respectively). The XGBoost-F-score model performed even worse compared to the three wrapper models, with an accuracy of only 0.615. These results indicated the potential of applying extracted GBM radiomics features for the prediction of low-grade glioma MGMT methylation status and hinted to the broader application of extracted features on both low- and high-grade glioma.

**Table 2:** Classification performances of the models on the LGG dataset.

Classifiers	Sensitivity	Specificity	Accuracy
**GA-RF**	**0.780**	**0.620**	**0.750**
GA-XGB	0.780	0.460	0.718
GA-SVM	0.840	0.23	0.718
XGB-Fscore	0.670	0.380	0.615

*RF* random forest, *XGB* XGBoost, *SVM* support vector machine, *XGB-Fscore* the F-score technique proposed by Le et al.

Model with the best performance is indicated in bold font.

### Comparisons with different Radiomics research for predicting the MGMT methylation status

To measure the effectiveness of our proposed method in predicting the MGMT methylation status, we compared the performance of our model against various published classifiers. As shown in Table 3, the MGMT prediction performances among different research^21,40–45^ vary between 0.67 and 0.925 in terms of accuracy. Even though Support Vector Machine^40^, L1-regularized neural networks^45^, and XGBoost algorithm^23^ yielded higher performances than others, the GA-RF method that we proposed in this study outperformed others, achieving the highest sensitivity, specificity, and accuracy. The result showed that the feature set identified using the GA-RF model may be better indicator in classifying MGMT methylation status.

**Table 3:** Comparisons between our models and other previous predictors of the MGMT methylation status in glioblastoma multiforme.

Study	Year	No. of features	Classifier	SN	SP	ACC
Le et al. ^23^	2020	9	XGBoost	0.88	0.887	0.887
Xi et al. ^21^	2018	63	Support vector machine	0.888	0.838	0.866
Levner et al. ^45^	2009	8	L1-regularized neural networks	0.854	0.9	0.877
Korfiatis et al. ^40^	2016	4	Support vector machine	0.803	0.813	N/A^a^
Crisi et al. ^42^	2020	14	Multilayer perception	0.75	0.85	N/A
Kanas et al. ^46^	2017	N/A	K-Nearest Neighbor	0.736	0.852	0.663
Sasaki et al. ^44^	2019	5	LASSO^b^	0.67	0.66	0.67
L Han et al. ^47^	2018	N/A	CRNN^c^	0.67	0.67	0.67
Ahn et al. ^43^	2014	N/A	Mann–Whitney U-test	0.563	0.852	N/A
**Our present study**	**2022**	**25**	**GA-RF**^**b**^	**0.894**	**0.966**	**0.925**

SN, sensitivity; SP, specificity; ACC, accuracy.

^a^“N/A” means that the information was not shown in the research.

^b^LASSO, least absolute shrinkage and selection operator; GA-RF, genetic algorithm-random forest. Bold font indicates the results of this study.

^c^Bi-directional convolutional recurrent neural network architecture.

## Discussion

In recent years, applications of radiomics have garnered a lot of attention because of their potential to provide meaningful interpretative and predictive information for guiding treatment strategies. Moreover, along with the exponential growth of imaging data, different ML and deep learning (DL) techniques have been applied to elucidate correlations between clinical symptoms and genetic characteristics to achieve more accurate prognoses and treatment responses. In this study, a hybrid ML-based radiomics feature selection model was developed to identify optimal radiomics feature sets and predict the MGMT promoter methylation status. We used a set of 704 radiomics features previously extracted from the TCGA-GBM dataset to test the performances of three different ML models using five-fold cross validation. The GA-RF algorithm in general outperformed the GA-XGB and GA-SVM (Table 1), showing that the devised genetic algorithm wrapper model was indeed capable of extracting important features for predicting the MGMT methylation status.

In cancer management, clinicians rely on tumor characteristics and grades to optimize treatment, including chemotherapy, radiation therapy, and surgical resection. It is common for patients to receive chemotherapy or radiation therapy for highly malignant tumors to minimize the tumor before its surgical removal. Therefore, an optimal and accurate radiomics feature set is important for clinicians in making decisions and guiding GBM treatments, as MGMT promoter methylation status may result in different decisions. In search of the solution, many different feature selection methods have been adopted, such as L1-regularized neural network^45^, least absolute shrinkage and selection operator (LASSO)^44^, F-scores^23^, or even using the Mann–Whitney U-test with Bonferroni correction to analyze the correlation between MGMT methylation statuses and quantitative imaging features, followed by the ROC curve analysis to choose the cutoff value for the presence of MGMT methylation^43^. To the best of our knowledge, most of the radiomics feature sets for classifying MGMT methylation statuses provided by other studies were merely based on one feature selection technique, and this is the first time the genetic algorithm-based hybrid feature selection approach has been applied for classifying MGMT methylation statuses in GBM. Therefore, this study aimed to test the feasibility of using the two-stage feature selection technique comprised of feature selection performed using the XGBoost algorithm followed by a GA wrapper model in picking radiomics feature subset that could effectively predict the MGMT methylation statuses. We observed that the adoption of the GA led to a radiomics feature set exhibiting accuracies higher than most of those reported in previous literature for the prediction of MGMT methylation statuses (Table 3). In addition, our findings showed that the inclusion of too few (F-score feature set) or too many features could both attribute to a lesser degree of prediction accuracy. As such, the GA represents a promising solution for the generation of highly-performing predictors, without a priori information about the optimal number of features to be included. The outstanding performances of the GA-based approach could be explained as follows. First, the GA is an evolutionary model in which the best individuals of the current generation are selected among the population to create the next generation with a potentially more powerful solution. Second, mutations and crossover occur by chance during the process of evolution, resulting in “fitter” offspring. By mimicking these natural selection phenomena, the GA-RF wrapper model was able to select the most important radiomics features based on the fitness function, crossover, and mutation processes.

By using the two-stage feature selection, our GA-RF algorithm generated an optimal subset of 22 radiomics features, including 17 textural features, three histogram-based features, one volume feature, and one intensity feature (Fig. 5). Interestingly, second-order statistical textural features, such as the gray-level size zone matrix (GLSZM) and gray-level run length matrix (GLRLM), appeared to be more frequently selected by the GA-RF classifiers and F-score, suggesting that these feature types are capable of better capturing the heterogeneous characteristics of the MGMT methylation status of GBM tumors. Indeed, many studies showed the potential of using textural features and gray-level tumor heterogeneity in some molecular characteristics, such as for classifying the 1p/19q-codeletion status^48^, IDH1 mutation classes^49^, or MGMT methylation status^40,45^. Computer-derived textural features were also shown to effectively classify GBM among other types of brain tumors, including low-grade gliomas and malignant glioneuronal tumors^48,50^. In addition, we noted that the majority of the GA-RF features were wavelet transform features (i.e., GLSZM and GLRLM) extracted by undecimated 3D wavelet transformations. Wavelet transformation is a technique by which the 3D image data can be split into various frequency components along three axes. Fine and coarse textural information extracted from wavelet-decomposed images can reflect the tumor heterogeneity at multiple scales^51^. A few studies also reported that wavelet features can act as important radiomics biomarkers to predict tumor phenotypes, since they are believed to have strong connections with tumor biological behaviors^52,53^. Our results suggested that wavelet-transform features could also play a crucial role in predicting the MGMT methylation status in GBM. In other words, the proposed method possesses great potential in “hunting” informative features for this prediction purpose.

Herein, we attempted to interpret the benefits of using texture- and histogram-based features in predicting the MGMT methylation status in a fundamental manner. Besides some major challenges associated with poor GBM prognoses such as late diagnosis, diffuse infiltration and pseudo-palisading necrosis, inter- and intra-tumor heterogeneity and the dynamic plasticity of cells were considered important characteristics that may exacerbate the ineffectiveness of GBM treatment and lower survival rates^54^. Therefore, texture- and histogram-based features could play critical roles to facilitate the GBM clinical diagnosis. Textural features reflecting spatial intensity correlations and distributions of voxels could help quantify the “multiregional variations” in blood flow, edema, necrosis, etc. For example, GLSZM_GLV (gray-level variance) reflects the intensity variance between homogeneous subregions within the enhancement area, while GLSZM_SZE (small-zone emphasis) is the distribution of short homogeneous zones in an image. On the other hand, histogram-based features illustrate the frequency distribution of intensity values that occur in an image. More specifically, these features quantify the statistical characteristics of an image and therefore reflect intratumor heterogeneity. Taken together, a combination of texture- and histogram-based features may boost the ability of machine learning models in discriminating methylated and unmethylated GBM tumors. Furthermore, our GA-RF feature sets also implied that to achieve an accurate prediction of the methylation status, a range of texture- and histogram-based features may be needed.

Although this proposed method has yielded promising results, there are still a few limitations. One of the limitations in radiomics studies is that imaging features extracted from a small number of patients often cause high dimensionality-related problems^55^. As the number of dimensions and data volume increases exponentially, sparser real differences may be obscured by random measurement noise^56,57^. This means that to gain statistical significance, high-dimensional feature space often demands a large number of samples. Second, this circumstance could also result in ML model overfitting when dealing with high-dimensional, small-sized datasets. We overcame the potentially overfitting problem by adopting cross-validation to make sure that the test portion does not interfere with the training process. We also note that even though the sample size is not very large, the features selected by the XGBoost feature selection algorithm and the published F-score study^23^ share a certain number of common features, indicating our two-step feature selection approach may have indeed selected meaningful features from this dataset. We also foresee that the approach can be further evaluated with the release of more processed radiomics datasets in the near future.

The application of the GBM radiomics features on the LGG dataset implies that the radiomics features extracted from one disease may be applicable to other very similar diseases. As shown in Results and Table 2, the evaluation of extracted feature set on the LGG dataset achieved an accuracy of 0.75. The results are very interesting since even though GBM and LGG are similar to each other in terms of preoperative radiomics techniques and low-level visual feature interpretation^58,59^, there are still slight differences between GBM and LGG in terms of their radiological appearance. In comparison with GBM, LGG tumors showed less blood–brain barrier disruption (resulting in less leak of contrast in the scan period) and little to no edema formation due to the slow growth rate of this kind of tumor, thus leading to low-density areas in MRI scans^25^. Reaching an accuracy of 0.75 on the MGMT methylation status prediction accuracy despite the fundamental differences between GBM and LGG indicated that some of the identified features may also be important in distinguishing the MGMT methylation statuses in LGG. We also tried to mix the GBM and LGG datasets into one mixed set and check whether we can identify common features among the two diseases. The application of the two-stage feature selection approach achieved 0.75 accuracy, 0.84 sensitivity, and 0.38 specificity. This showed that such mixing effort does not lead to better outcome, as seen in the much-reduced specificity. We plan to continue investigating the “common feature for very similar disease” issue in our future work.

## Conclusions

To the best of our knowledge, this is the first study implementing an ML-incorporated GA model for predicting the MGMT methylation status. Results of this study showed that our proposed method could noninvasively predict the MGMT methylation status with a superior performance compared to existing methods. The model with the highest performance (GA-RF) was tested on an independent dataset, which demonstrated that the model may be generalized to similar diseases. Predicting the MGMT methylation status by this state-of-the-art model could benefit clinical decision-making by accommodating treatment strategies for patients with GBM even before surgery.

## Data Availability

The source code of the GA-RF approach is available at Github public repository (https://github.com/thiduyendo/GA-ML). The data analyzed in this study were all downloaded from The Cancer Imaging Archive (TCIA) website (https://www.cancerimagingarchive.net/tcia-analysis-results/).
